# Antibodies to histone in the pediatric population: a retrospective chart review

**DOI:** 10.1186/s12969-023-00821-y

**Published:** 2023-04-25

**Authors:** C. Matt Justice, Terry L. Moore

**Affiliations:** Division of Rheumatology and Pediatric Rheumatology, Saint Louis University School of Medicine, St. Louis, MO USA

**Keywords:** Histone, Pediatric, Systemic lupus erythematosus(SLE), Juvenile idiopathic arthritis(JIA), Drug induced lupus(DILE), Uveitis

## Abstract

**Background:**

Antibodies to histone have been associated in the adult literature with systemic lupus erythematosus(SLE) and drug induced lupus(DILE). Little data is available regarding the spectrum of pathology that antibodies to histone encompass in the pediatric population. Prior studies suggest an association with SLE, juvenile idiopathic arthritis(JIA), uveitis and linear scleroderma.

**Methods:**

Patient charts were reviewed that contained positive anti-histone antibody testing during a consecutive three year period. Patient diagnosis along with the presence of: anti-histone antibody titer, ANA, and the presence of other autoantibodies to SSA, SSB, Sm, RNP, dsDNA and chromatin were obtained. The frequency of SLE, JIA and DILE was further investigated in specific subsets.

**Results:**

139 individual charts were reviewed containing 41 different diagnoses. The most common diagnosis was hypermobility arthralgia with 22 patients. The most frequent rheumatologic diagnosis was JIA(nonsystemic) with 19. 13 patients in this study were diagnosed with SLE and 2 with DILE. 18 patients had other autoantibody production, of these, 11 had SLE or DILE. Only one of 62 patients with a weak antihistone antibody titer(1.0-1.5) was diagnosed with SLE. When strong titers are present(> 2.5), the antihistone antibody test was associated with a greater than 50% incidence of an underlying rheumatologic disease and ten times higher incidence of SLE than a weak titer. In regards to the frequency of SLE, there was a statistically significant difference between weak and moderate titers and between weak and strong titers.

**Conclusion:**

The presence of anti-histone antibody was observed in a variety of diagnoses in the pediatric population. Overall, the presence of anti-histone antibodies appears to have poor diagnostic utility for any specific condition. However, diagnostic utility for SLE does appear to improve with higher titers, when combined with other autoantibody positivity. Strength of titer did not appear to be a factor for JIA, but was the most frequently observed rheumatologic disease in this study.

## Background

Antibodies to histone have been described in the adult literature in patients with SLE and DILE. Little data is available currently regarding the spectrum of pathology that antibodies to histone encompass in the pediatric population. Prior studies suggest an association with JIA, uveitis, and linear scleroderma(LS) in addition to SLE. At present, anti-histone antibody testing is readily available and is frequently performed as part of the subsequent workup for ANA positivity, JIA, SLE and other rheumatologic diseases. Positive results are frequent, often with unclear significance.

Anti-histone antibodies were first detected in SLE in 1960 and subsequently re-demonstrated in 1971 and 1976[1]. In 1978, a study showed higher incidence of anti-histone antibodies in patients with DILE versus SLE [2]. It was suggested that anti-histone antibodies in SLE may have some correlation with disease activity [1]. Histones are basic DNA binding proteins and are among the more common targets of autoantibodies seen in patients with SLE. Individual histones H1, H2A, H2B, H3, H4 have been identified and studied within the context of SLE, but their clinical value is limited [3]. Antibodies to histone detected by ELISA were present in 100% of 20 patients with DILE, 42% of 60 patients with SLE and 15% of 20 adults with rheumatoid arthritis [4].

Although adult data regarding SLE may be applicable to pediatrics, studies are lacking in regards to antihistone antibodies in the pediatric population. It was shown in a pediatric and adolescent-onset SLE population that anti-histone antibodies correlated significantly with leukopenia, hemolytic anemia, and dsDNA antibody titers [5]. There is also a suspected association between anti-histone antibodies and JIA. Antibody to histone H1 was found in 42% of the JIA serum samples [6]. Another study suggested that anti-histone antibodies seen in pediatric patients with JIA may have different histone selectivity than in adult SLE. This study showed a predominance of anti-H1 and anti-H5 antibodies and relative absence of antibodies binding to core histones in JIA, in contrast to findings in adult SLE [7].

ANA positivity in JIA has long been associated with chronic anterior uveitis. An association with antihistone antibodies has been proposed. One study showed that 58 (48%) of 121 patients with JIA tested positive for anti-histone antibodies. Twenty-eight of 30(93%) of patients with JIA with uveitis had antihistone antibodies while only 30(33%) of 91 patients without uveitis had anti-histone antibodies. This same study also suggests that anti-H3 specific histone antibodies correlated with uveitis in the JIA population [8, 9]. More recent studies have also shown higher titer anti-histone antibodies as a risk factor development of uveitis in JIA [10].

In one small study of mostly pediatric patients, results showed a high prevalence of anti-histone antibodies in LS. Ten of 14(71%) of pediatric patients with LS of the torso and/or extremities had antibodies to histone. Five of 11(45%) of pediatric patients in the study with frontoparietal LS were positive [11].

The purpose of this study was to further investigate the frequency of different rheumatologic diseases in the pediatric population with positive testing for anti-histone antibodies. This could potentially allow for better practical application of the test in clinical practice.

## Methods

All charts from the Cardinal Glennon Children’s Hospital Pediatric Rheumatology clinic from 1/1/2016 to 12/31/2019 with positive anti-histone antibody tests were reviewed. Frequently, these were evaluations for possible systemic lupus erythematosus or inflammatory arthritis with positive ANA testing. In addition to the anti-histone antibody titer, age, diagnostic codes, and the presence of ANA, anti DS-DNA, chromatin, SSA, SSB, Sm and RNP antibodies were recorded. In the instances where multiple auto-antibody profiles were available, the most recent was used. Charts were manually reviewed for the treating Rheumatologist’s most recent diagnosis which was recorded and used for statistical analysis. In the case of multiple diagnoses, each relevant diagnosis was recorded. Patients were allowed to have more than one diagnosis. Patients whose charts specifically noted being on medications associated with DILE, but did not have clinical manifestations were considered as possible drug induced autoantibody formation and not DILE.

Diagnoses were also grouped into the category of rheumatologic diagnoses and autoimmune diagnoses. Rheumatologic diagnoses were defined as SLE, Sjogren’s syndrome, chronic recurrent multifocal osteomyelitis(CRMO), inflammatory bowel disease(IBD)/IBD arthritis, Behcet’s, rheumatoid arthritis, JIA(all subtypes except systemic), systemic JIA, DILE, uveitis, psoriasis/psoriatic arthritis, undifferentiated connective tissue disease, inflammatory myopathy, linear scleroderma(LS) and Henoch Schonlein Purpura. Autoimmune diagnoses were defined as autoimmune hepatitis, autoimmune thyroid disease, celiac disease and type 1 diabetes in addition to the previously mentioned rheumatologic diagnoses. This was done to allow calculation of a positive predictive value for a positive anti-histone antibody test in reference to any autoimmune or rheumatologic disease given the low incidence of specific diagnoses observed in the population.

Weakly positive anti-histone titers were defined as a level from 1.0 to 1.5 units. Moderate titers were defined as 1.6–2.5 units and strongly positive titers defined as greater than 2.5 units in accordance with how results are reported back to the clinician from LabCorp. These are the standard cut-offs used by LabCorp. Anti-Histone antibody tests were performed by LabCorp using an IgG class ELISA test. Data regarding individual histone subtypes is not performed by LabCorp and was not available regarding the patients included in this study.

Positive predictive value was calculated for SLE, JIA(non-systemic), DILE, and any rheumatologic or autoimmune diagnosis. This calculation was made using the standard formula for positive predictive value (true positives divided by total positive tests [true positive plus false positive]). The clinical diagnosis (if present) from chart review was considered a true positive for purposes of calculating positive predictive value. This was then divided by the number of total positive anti-histone antibody titers within each specific subset analyzed. Subsets included patients with positive anti-histone antibodies in conjunction with other autoantibodies, along with patients with low titer anti-histone antibodies and negative ANA testing without other autoantibodies present.

A basic 2 × 2 Chi-square calculation was used to compare weak, moderate, and strong titers of antihistone antibodies in regards to the frequency of autoimmune disease in general and SLE. Specifically, weak titer was compared to moderate titer, moderate titer compared to high titer and weak titer compared to high titer.

This study was approved by the Saint Louis University Institutional Review Board, protocol #30,713.

## Results

139 individual charts were reviewed. There were 41 different diagnoses present in the study group. The most common diagnosis recorded was hypermobility arthralgia which was present in 22 patients. This was followed by arthralgias(without JIA) in 21 patients. The most frequent rheumatologic diagnosis was JIA(non-systemic) with 19. The other diagnoses are enumerated in Table 1.

**Table 1:** Observed Diagnoses in Patients with Positive Anti-Histone Antibody Test.

Diagnosis	Number
Hypermobility Arthralgia	22
Arthralgia(not JIA)	21
JIA(All subtypes except systemic)	19
Suspected Drug Induced Auto-antibodies(without lupus)	17
ANA positive(without other diagnosis)	14
Systemic Lupus erythematosus	13
Raynaud’s Phenomenon	7
Inflammatory Bowel Disease	5
Uveitis(without JIA)	4
Acrocyanosis	4
Chronic Recurrent Multifocal Osteomyelitis	3
Linear Scleroderma	3
Recurrent Fevers	3
Sjogren’s	2
IBD related Arthritis	2
Epstein Barr Virus	2
Ehler’s Danlos	2
Drug Induced Lupus	2
Systemic JIA	2
Elevated CK(without inflammatory myopathy)	2
Autoimmune Thyroid Disease	2
Undifferentiated Connective Tissue Disease	2
Fibromyalgia / Amplified Pain Syndrome	2
Complex Regional Pain Syndrome	2
Type 1 Diabetes Mellitus	1
Behcet’s	1
Celiac Disease	1
Rheumatoid Arthritis	1
Alopecia	1
Foot Swelling	1
SSA positive(without Sjogren’s)	1
Psoriasis(without arthritis)	1
Dupuytren’s Contractures	1
Inflammatory Myositis	1
Psoriatic Arthritis	1
Myalgia	1
Autoimmune Hepatitis	1
Sever’s Disease	1
Osgood-Schlatter Disease	1
Rash	1
Henoch-Schonlein Purpura	1

A total of 13 patients in this study were diagnosed with SLE and 2 with DILE. 56 patients had an underlying rheumatologic diagnosis and 61 were considered to have had an autoimmune diagnosis including autoimmune hepatitis, autoimmune thyroid disease, type 1 diabetes, and celiac disease. Positive predictive values for SLE, DILE and JIA were all very low (Table 2).

**Table 2:** Positive Predictive Values for Anti-Histone Antibody Tests

Subset	Number (n, total 139)	Positive Predictive Value
Systemic Lupus erythematosus	13	0.094
Drug Induced Lupus	2	0.014
JIA(all subtypes except systemic)	19	0.136
Any Rheumatologic Diagnosis	56	0.403
Any Autoimmune Diagnosis	61	0.439

Only 69 patients out of 139 had a + ANA in addition to a positive anti-histone antibody level. 70 patients were ANA negative. 18 patients had other autoantibody production in addition to anti-histone antibodies. Of these patients with other autoantibody production, 14 had an autoimmune diagnosis and 11 had SLE or DILE (Table 3).

**Table 3:** Specific Subsets (Positive predictive value shown in Parentheses)

Subset	Number	Rheumatologic Diagnosis	Systemic Lupus	Drug Induced Lupus	JIA(nonsystemic)
Other Autoantibodies Present**	18	14(0.778)	10(0.566)	1(0.0556)	1(0.0556)
Weak + antihistone***, ANA negative, no other Autoantibodies**	34	10(0.294)	0	1(0.029)	5(0.147)

**SSA, SSB, Chromatin, Double Stranded DNA, Smith, RNP

***Anti-histone titer 1.0-1.5

34 patients in the study had weakly positive anti-histone antibodies, negative ANA titer and no other autoantibody production. Of these 34, 10 had a rheumatologic diagnosis, 1 had a diagnosis of DILE and none had a diagnosis of SLE (Table 3).

When separated out by strength of anti-histone antibody titer, 62 total patients had low positive antihistone antibody titers. 34 total patients had moderate anti-histone antibody titers and 43 total patients had strongly positive anti-histone antibody titers (Table 4). Of those with weak positive titers, 19 out of 62 carried a rheumatologic diagnosis. 13 out of 34 patients with a moderate titer of histone antibodies had a rheumatologic diagnosis and 24 of 43 with strongly positive histone antibody titers had a rheumatologic diagnosis. Of note, the lowest histone antibody titer observed in a patient with SLE was 1.3 in this study.

**Table 4:** Rheumatologic Diagnosis by Strength of Titer(Positive predictive value)

Titer	Number	Rheumatologic Disease	Autoimmune Disease	Systemic Lupus	Drug Induced Lupus	JIA(nonsystemic)
Weak(1.0-1.5)	62	19(0.306)	19(0.306)	1(0.016)	1(0.016)	10(0.161)
Moderate(1.6-2.5)	34	13(0.382)	15(0.441)	5(0.147)	0	1(0.029)
Strong(> 2.5)	43	24(0.558)	27(0.628)	7(0.163)	1(0.023)	8(0.186)

Only one of 62 patients with a low titer histone antibody in this study had a diagnosis of SLE. This increased to 5 out of 34 and 7 out of 43 in the moderate and strong titer groups respectively. A patient with a strong titer anti-histone antibody titer was approximately ten times more likely to have SLE than a patient with low titer in this study population and about twice as likely to have an autoimmune disease.

The incidence of JIA was highest in the strong titer group. The lowest incidence of JIA was in the moderate titer group.

In regards to the frequency of overall autoimmune disease, there was no significant difference between weak and moderate titers (p-value 0.187) or moderate and strong titers (p-value 0.102). There was a statistically significant increase between weak and strong titers of anti-histone antibodies (p-value 0.000028).

In regards to the frequency of SLE, there was a statistically significant increase between weak and moderate titers (p-value 0.0113) and between weak and strong titers (p-value 0.0053), but not between moderate and strong titers(p-value 0.850).

## Discussion

Anti-histone antibodies are seen in a variety of conditions both rheumatologic and non-rheumatologic in the pediatric population. The two most frequent diagnoses were hypermobility arthralgia and arthralgia (without JIA). This would suggest that the anti-histone antibody is routinely present in patients who have joint pain, but do not have underlying JIA or SLE.

Positive predictive values calculated for SLE, JIA and DILE were low. The most common rheumatologic diagnosis encountered was JIA(all subtypes excluding systemic), which had a positive predictive value of only 0.136. Even when combining all autoimmune conditions, the percentage of patients with a positive anti-histone antibody test and an underlying autoimmune disease was still less than half at 43.9%. The strength of titer of anti-histone antibodies may be a factor for both the overall occurrence of autoimmune disease and SLE. There were statistically significant differences between weak and strong titers. Strength of titer did not appear to be a factor for JIA in this study.

The anti-histone antibody test did perform differently when specifically examined in the group of patients who had other autoantibodies. 78%(14/18) of patients with other autoantibody production including antibodies to SSA, SSB, Sm, RNP, Chromatin, and dsDNA did have an underlying rheumatologic diagnosis. Of these, 10 were diagnosed with SLE. This suggests that the presence of antibodies to other extractable nuclear antigens in addition to histone increases the likelihood of a diagnosis of SLE.

The classic association with DILE and anti-histone antibodies from previous adult literature was seldom observed in this population. Only 2/139 patients in this study were diagnosed with DILE. Seventeen patients in this study had positive anti-histone antibody tests without features of SLE or DILE, but were noted to be on medications associated with DILE. Data about the frequency of specific medications was not collected in this study but does provide an opportunity for further investigation.

Patients with low titer anti-histone antibodies, negative ANA, and no other autoantibody production had a low incidence of underlying autoimmune diseases (10/34 patients). This was less than one third, but not totally negligible. Five of these patients were diagnosed with JIA and one with SLE.

JIA was the most frequent rheumatologic disease seen in this study. It’s possible this trend could be secondary to the increasing use of TNF inhibitors leading to autoantibody formation or just a reflection of higher prevalence of JIA in general when compared to SLE in the pediatric population.

Patients in this study were taken from a population undergoing evaluation in a pediatric rheumatology clinic. The results may not be representative of the general population.

## Conclusion

Anti-histone antibodies were observed in a variety of diagnoses in the pediatric population, many of which are considered benign or not rheumatologic. Most frequently, anti-histone antibodies were present in patients without underlying autoimmunity, especially at weak titers. DILE was uncommon in this study with only two cases. Not surprisingly, the positive predictive value of the anti-histone antibody test for SLE did improve in the subpopulation that demonstrated other autoantibody production(antibodies to SSA, SSB, Sm, RNP, Chromatin, and dsDNA). Similar to the ANA test, positive results were present in significant numbers of patients without underlying autoimmunity and testing should be reserved for cases in which there is a high underlying clinical suspicion of autoimmune disease such as SLE. This further exemplifies that the diagnosis of rheumatologic disease requires more than abnormal auto-antibody testing.

Further research is still needed to investigate if there is any significance to the presence of anti-histone antibodies and clinical phenotype in JIA. Association with uveitis has been proposed previously, but other factors may also exist. The increasing availability and sharing of patient data within pediatric rheumatology might make this possible.

The overall results of this study show that the presence of anti-histone antibodies in the pediatric population alone are a poor predictor of any specific condition, especially at weakly positive titers.

## Data Availability

The datasets used and/or analyzed during the current study are available from the corresponding author on reasonable request.

## Abbreviations

SLE: systemic lupus erythematosus
DILE: drug induce lupus
JIA: juvenile idiopathic arthritis
LS: linear scleroderma
CRMO: chronic recurrent multifocal osteomyelitis
IBD: inflammatory bowel disease
HSP: Henoch Schonlein Purpura
